# Action Seniors! - secondary falls prevention in community-dwelling senior fallers: study protocol for a randomized controlled trial

**DOI:** 10.1186/s13063-015-0648-7

**Published:** 2015-04-10

**Authors:** Teresa Liu-Ambrose, Jennifer C Davis, Chun Liang Hsu, Caitlin Gomez, Kelly Vertes, Carlo Marra, Penelope M Brasher, Elizabeth Dao, Karim M Khan, Wendy Cook, Meghan G Donaldson, Ryan Rhodes, Larry Dian

**Affiliations:** Aging, Mobility, and Cognitive Neuroscience Laboratory, Djavad Mowafaghian Centre for Brain Health, University of British Columbia, 212-2177 Wesbrook Mall, Vancouver, BC V6T 1Z3 Canada; Centre for Clinical Epidemiology and Evaluation, Vancouver Coastal Health Research Institute & University of British Columbia, 7th Floor, 828 West 10th Avenue, Research Pavilion, Vancouver, BC V5Z 1M9 Canada; School of Pharmacy, Memorial University of Newfoundland, Health Sciences Centre, St. John’s, NL A1B 3V6 Canada; Centre for Hip Health and Mobility, Vancouver Coastal Health Research Institute & University of British Columbia, 2635 Laurel St, Vancouver, BC V5Z 1M9 Canada; St Paul’s Hospital, University of British Columbia, 1081 Burrard Street, Vancouver, BC V6Z 1Y6 Canada; Vancouver Coastal Health Research Institute, Room 3665, 910 West 10th Avenue, Vancouver, BC V5Z 1M9 Canada; Behavioural Medicine (BMED) Lab, School of Exercise Science, Physical and Health Education, University of Victoria, 3800 Finnerty Road, Victoria, BC V8P 5C2 Canada

**Keywords:** Otago Exercise Program, Falls, Resistance Training, Executive Functions, Older Adults

## Abstract

**Background:**

Falls are a ‘geriatric giant’ and are the third leading cause of chronic disability worldwide. About 30% of community-dwellers over the age of 65 experience one or more falls every year leading to significant risk for hospitalization, institutionalization, and even death. As the proportion of older adults increases, falls will place an increasing demand and cost on the health care system. Exercise can effectively and efficiently reduce falls. Specifically, the Otago Exercise Program has demonstrated benefit and cost-effectiveness for the primary prevention of falls in four randomized trials of community-dwelling seniors. Although evidence is mounting, few studies have evaluated exercise for secondary falls prevention (that is, preventing falls among those with a significant history of falls). Hence, we propose a randomized controlled trial powered for falls that will, for the first time, assess the efficacy and efficiency of the Otago Exercise Program for secondary falls prevention.

**Methods/Design:**

A randomized controlled trial among 344 community-dwelling seniors aged 70 years and older who attend a falls prevention clinic to assess the efficacy and the cost-effectiveness of a 12-month Otago Exercise Program intervention as a secondary falls prevention strategy. Participants randomized to the control group will continue to behave as they did prior to study enrolment. The economic evaluation will examine the incremental costs and benefits generated by using the Otago Exercise Program intervention versus the control.

**Discussion:**

The burden of falls is significant. The challenge is to make a difference – to discover effective, ideally cost-effective, interventions that prevent injurious falls that can be readily translated to the population. Our proposal is very practical – the exercise program requires minimal equipment, the physical therapist expertise is widely available, and seniors in Canada and elsewhere have adopted the program and complied with it. Our innovation includes applying the intervention to a targeted high-risk population, aiming to provide the best value for money. Given society’s limited financial resources and the known and increasing burden of falls, there is an urgent need to test this feasible intervention which would be eminently ready for roll out.

**Trial registration:**

ClinicalTrials.gov Protocol Registration System: NCT01029171; registered 7 December 2009.

## Background

Falls are a common geriatric syndrome [1] and are the third leading cause of chronic disability worldwide [2]. Falls impose significant risk for hospitalization, institutionalization, and even death [3-5]. About 30% of community-dwellers over the age of 65 experience one or more falls every year [6], with half of these seniors experiencing recurrent falls. With the proportion of older adults increasing, falls will continue to place an increasing health and economic burden on the public health system.

Exercise can effectively reduce falls. Specifically, New Zealand researchers designed a physical therapist-delivered, progressive home-based strength and balance training program tailored for seniors [7-11]. This intervention – the Otago Exercise Program (OEP) – has demonstrated benefit in four randomized trials of community-dwelling seniors selected based on age alone (that is, ≥ 80 years old) [7-11]. Only one of these four trials designated falls as the primary outcome [12] while the others focused on measures of falls risk. Hence, the OEP qualifies as primary falls prevention (that is, preventing falls among those without a history of falls). The Cochrane Collaboration [13] explicitly identifies the OEP as the exercise training program with the strongest evidence for falls prevention. Although the OEP is the exercise training program with the strongest evidence for primary falls prevention [13], no randomized controlled trial (RCT) powered for falls has evaluated the efficacy of the OEP as a secondary falls prevention (that is, preventing falls among those with a history of falls) strategy. Hence, a rigorously designed RCT with falls as the primary outcome is an essential next step to determine the role of OEP in preventing falls among senior men and women with a significant history of falls. Previous research has demonstrated that the best value for money of various falls prevention strategies comes from targeting high-risk groups [14].

Improved physiological function is the generally accepted mechanism underlying the effectiveness of the OEP in reducing falls [8]. However, in a meta-analysis of four OEP randomized trials, falls were significantly reduced by 35% while postural sway significantly improved by only 9% and there was no significant improvement in knee extension strength [11]. Hence, the OEP may reduce falls via mechanisms other than improved physiological function. Specifically, we have demonstrated proof-of-concept data suggesting that improved cognitive function may be a very important mechanism by which the OEP reduces falls [15].

Within the multiple domains of cognitive function, reduced executive functions are associated with falls [16-20]. Executive functions are higher order cognitive processes that control, integrate, organize, and maintain other cognitive abilities [21]. Executive functions decline substantially with aging [22]. Importantly, reduced executive functions are prevalent among healthy, community-dwelling seniors with intact global cognitive function (that is, Mini-Mental State Examination (MMSE) score ≥24/30) [23,24]. This is not surprising given that many of the pathological changes (for example, white matter lesions) associated with reduced executive functions are prevalent but clinically silent [25]. Our proof-of-concept study provided preliminary evidence that the OEP may improve executive functions in senior fallers [15]. Given the association between executive functions, exercise, and falls, we hypothesize that improved executive functions may be an important mechanism by which exercise reduces falls. However, this hypothesis is yet to be tested. Furthermore, our proof-of-concept study did not have the sample size to explore whether the observed change in cognitive function was a mediator of the benefit of the OEP.

Thus, we propose a 12-month RCT among community-dwelling seniors aged 70 years and older who attend a secondary falls prevention clinic to assess the efficacy and the cost-effectiveness of the OEP as a secondary falls prevention strategy. Further, we aim to explore the relative importance of both physiological and cognitive factors to falls reduction. Given the immense health and financial burden imposed by falls, our proposed RCT could have significant impact on the health of Canadian seniors and the Canadian health care system.

## Methods

### Design

**Figure 1:**
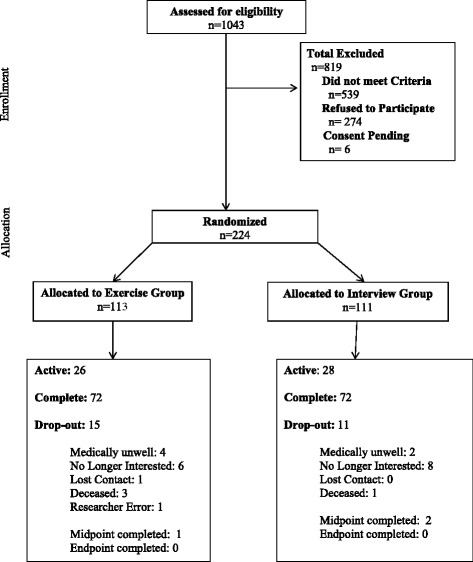
**Overview of the flow of participants through the Action Seniors! trial.**

We propose a RCT of 344 community-dwelling senior with a history of falls (that is, one or more falls in the past 12 months), aged 70 and older. Participant randomized to the OEP intervention group will receive the intervention for 12-months. There will be three measurement sessions with monthly monitoring (Figure 1).

### Setting

All participants will be recruited from the Falls Prevention Clinic at Vancouver General Hospital (www.fallclinic.com).

### Participants

All participants attending the Falls Prevention Clinic have sustained one or more falls in the past 12 months. Referrals to the Falls Prevention Clinic are from health care professionals (for example, physicians) for those who sought medical attention for their fall. Patients who attend the Falls Prevention Clinic receive falls risk factor assessment followed by a comprehensive geriatric assessment. The Falls Prevention Clinic care pathway is based on the American Geriatrics Society/British Geriatrics Society/American Academy of Orthopedic Surgeons Falls Prevention Guidelines [26] (which is hereafter referred to as “standard of care”).

Charts from the clinic will be reviewed on a weekly basis to identify eligible participants. Those who appear eligible based on detailed chart review will be mailed an information package and asked to call a research assistant if they are interested in participating in the study. When phone contact generates a person’s agreement to participate, a research assistant will follow-up with a home visit. During this home visit, the consent form will be reviewed. Once written informed consent is obtained, the research assistant will complete the baseline assessment. Upon completion of the assessment, the research assistant who will remain blinded to group allocation will contact the research coordinator who will access the central randomization service to reveal the treatment allocation.

### Eligibility

#### Inclusion criteria

1. Adults ≥70 years referred by a medical professional to the Falls Prevention Clinic as a result of seeking medical attention for a non-syncopal fall in the previous 12 months
2. Understands, speaks, and reads English proficiently
3. MMSE [27] score ≥24/30
4. A Physiological Profile Assessment (PPA; Prince of Wales Medical Research Institute, Sydney, Australia) [28] score of at least 1.0 standard deviation above age-normative value
  or
  Timed Up and Go (TUG) test [29] performance of greater than 15 seconds
  or
  one additional non-syncopal fall in the previous 12 months
5. Expected to live greater than12 months (based on the geriatricians’ expert opinion);
6. Living in the Greater Vancouver area
7. Community-dwelling (that is, not residing in a nursing home, extended care unit, or assisted-care facility)
8. Able to walk 3 meters with or without an assistive device
9. Able to provide written informed consent

#### Exclusion criteria

1. Previously diagnosed with or suspected (by the geriatrician) to have neurodegenerative disease (for example. Parkinson’s disease)
2. Previously diagnosed with or suspected (by the geriatrician) to have dementia (of any type)
3. Had a stroke
4. Have a history indicative of carotid sinus sensitivity (that is, syncopal falls)

Ethical approval has been obtained from the Vancouver Coastal Health Research Institute (V10-70171, 11 May 2004) and the University of British Columbia’s Clinical Research Ethics Board (H04-70171, 11 May 2004).

### Power calculation

The primary outcome is self-reported number of falls over the 12-month follow-up period. The sample size calculation employs a negative binomial regression model [30] to account for the overdispersion typical of falls data. Assuming an average fall rate in the control group of 1.0 falls per year, an average follow-up of 0.9 years and an overdispersion parameter, φ, of 1.6, we require 163 seniors per group to have 80% power to detect a 35% relative reduction in fall rate (that is, 1.0 versus 0.65 falls per year). To accommodate a complete loss to follow-up rate of 5% (that is, no fall diaries returned) we will recruit a total of 344 seniors (that is, 172 per group). The estimate of the control fall rate comes from the pooled analysis of four trials in a similar population [11]. The estimate of the overdispersion parameter comes from analysis of the data in Table two of Shumway-Cook [31] which yields φ = 1.6. The estimate for the average length of follow-up is based on our previous proof-of-concept study conducted locally in the same patient population in Greater Vancouver [15,32]. Only one of 74 participants returned no fall diaries so our estimate of a 5% complete loss to follow-up rate is conservative [32].

### Measurements

Baseline measurements will be obtained prior to randomization. There will be three measurement sessions: baseline, 6 months, and 12 months.

#### Falls prevention clinic visit

The measurements listed below are acquired as part of the Falls Prevention Clinic visit and will be collected as the participants' baseline values upon informed consent.

##### Anthropometry

Standing height is measured as stretch stature to 0.1 cm per standard protocol. Weight will be measured to 0.1 kg on a calibrated digital scale.

##### Geriatrician examination

All patients undergo a comprehensive geriatrician assessment based on the American Geriatrics Society/British Geriatrics Society/American Academy of Orthopedic Surgeons Falls Prevention Guidelines [26].

##### General health, falls history, and socioeconomic status

General health, falls history in the last 6 months [33], and socioeconomic status are ascertained by questionnaires.

##### Global cognitive function

Global cognitive function is assessed using both the MMSE [27] and the Montreal Cognitive Assessment (MoCA) [34]. The MoCA is a brief 30-point screening tool for mild cognitive impairment [34] with high sensitivity and specificity. Specifically, it is more sensitive than the MMSE in detecting mild cognitive impairment. Using a cut-off score of 26, the MMSE had a sensitivity of 18%, whereas the MoCA detected 90% of individuals with mild cognitive impairment [34].

##### Balance and mobility

General balance and mobility is assessed with the: 1) Short Physical Performance Battery [35]; and 2) the TUG test [29]. For the Short Physical Performance Battery, participants are assessed on performances of standing balance, walking, and sit-to-stand. Each component is rated out of four points, for a maximum of 12 points. Poor performance on this scale predicts subsequent disability [35]. For the TUG test, participants are instructed to rise from a standard chair, walk a distance of 3 meters, turn, walk back to the chair and sit down. A TUG performance time of ≥13.5 seconds correctly classified persons as fallers in 90% of cases [36].

##### Physiological falls risk

We use the PPA [28] to assess physiological falls risk. The PPA is a valid and reliable tool for falls risk assessment. Based on the performance of five physiological domains (postural sway, hand reaction time, quadriceps strength, proprioception, and edge contrast sensitivity), the PPA computes a falls risk score for each individual and this measure has a 75% predictive accuracy for falls in older people [28]. A PPA z-score of 0 to 1 indicates mild risk, 1 to 2 indicates moderate risk, 2 to 3 indicates high risk, and 3 and above indicates marked risk [37].

##### Mood

We use the 15-item Geriatric Depression Scale [38,39] to screen for depression. The Geriatric Depression Scale specifically assesses for depressed mood in older people and a score of 5 and greater indicates depression [39].

##### Co-morbidity

The Functional Co-morbidity Index was calculated to estimate the degree of co-morbidity associated with physical functioning [40].

##### Instrumental Activities of Daily Living scale

The Lawton and Brody [41] Instrumental Activities of Daily Living Scale screens for impaired instrumental activities of daily living. This scale subjectively assesses ability to telephone, shop, prepare food, housekeep, do laundry, handle finances, be responsible for taking medication, and determining mode of transportation.

#### Baseline home visit

The following additional measures will be acquired during the home visit when written consent is obtained. The maximum time lag between the baseline Falls Prevention Clinic visit and the home visit is 1 month.

##### Falls-related self-efficacy

Falls-related self-efficacy will be assessed by the Activities-Specific Balance Confidence (ABC) Scale. The 16-item ABC Scale [42] assesses falls-related self-efficacy with each item rated from 0% (no confidence) to 100% (complete confidence). The ABC Scale score is correlated with other measures of self-efficacy, distinguishes between individuals of low and high mobility, and corresponds with balance performance measures [43,44].

##### Physical activity level

Current physical activity level will be assessed by the valid and reliable Physical Activities Scale for the Elderly questionnaire [45,46]. This 12-item scale measures the average number of hours per day spent participating in leisure, household, and occupational physical activities over the previous 7-day period.

##### Executive functions

There is no unitary executive function – rather, there are distinct processes. Thus, no single measure of executive function can adequately tap the construct in its entirety. Within the context of our proposal, we refer to work by Miyake and colleagues [47] who identified three key executive processes: 1) set shifting; 2) updating (or working memory); and 3) selective attention and conflict resolution (or response inhibition). Set shifting requires one to go back and forth between multiple tasks or mental sets [47]. Updating involves monitoring incoming information for relevance to the task at hand and then appropriately updating the informational content by replacing old, no longer relevant information with new incoming information. Conflict resolution involves deliberately inhibiting dominant, automatic, or prepotent responses. We will assess: 1) set shifting using the Trail Making Test (Part A and B) [48]; 2) updating (that is, working memory) using the verbal digits forward and backward test [49]; and 3) response inhibition using the Stroop Colour-Word Test [50]. These standardized neuropsychological tests are sensitive to age- [48,51] and intervention-related changes [52-56]. Information processing speed will be indexed using the Digit Symbol Substitution Test [57]. For this task, participants are first presented with a series of numbers (1 to 9) and their corresponding symbols. They are asked to draw the correct symbol for any digit placed randomly in pre-defined series in 60 seconds. A higher number of correct answers in this time period indicates better executive functions and processing speed.

##### Verbal fluency

Defined as the rate at which an individual can generate words, verbal fluency will be assessed using both the FAS test (which assesses phonemic verbal fluency) and the animal naming test (which assesses semantic verbal fluency) [48]. For the FAS verbal fluency test, participants will be asked to verbally generate as many words (excluding proper names) as they can starting with the letters “F”, “A” and “S”, each in 60 seconds [48]. The total number of words generated for all three letters will be used as the measure of performance. For the animal naming test, participants will be asked to generate a list of animal names in 60 seconds [48].

##### Health-related quality of life

We will evaluate health-related quality of life using Euro-Qol-5D three level (EQ-5D-3 L) [58]. The EQ-5D ascertains health status according to the following domains: mobility, self-care, usual activities, pain, and anxiety/depression. We will calculate quality-adjusted life years using the weightings from each instrument to compare differences in the incremental cost-effectiveness ratios.

#### Monthly measurement

The following measures will be collected monthly by telephone: 1) current physical activity level as assessed by the Physical Activities Scale for the Elderly questionnaire; and 2) health-related quality of life as assessed by the Short Form 6D [59], EuroQol EQ-5D-3 L [58], and Health Utilities Index Mark 3 [60]. Strategies to promote adherence to the OEP exercises during these monthly phone calls will also occur.

Through monthly calendars and diaries, participants will be asked to provide the following information: 1) falls and adherence to the OEP (ascertainment of falls and adherence to the OEP will be documented on monthly calendars); and 2) health care resource utilization and costs (participants will complete monthly health care resource use diaries over the 12-month study period).

### Randomization

Participants will be randomly assigned (1:1) to either the OEP (plus standard of care) group or the standard of care (control) group. The randomization sequence will be stratified by: 1) sex, as falls rate is different between men and women; and 2) geriatrician (LD and WC), as standard of care delivery may differ between physicians. Permuted blocks of varying size (for example, 2,4,6) will be employed. To ensure concealment of the treatment allocation, the randomization sequences will be generated and held by a central Internet randomization service.

### Planned trial interventions

#### Otago Exercise Program intervention

The OEP is an individualized home-based balance and strength retraining program [8,61]. It consists of the following strengthening exercises: knee extensor (4 levels), knee flexor (4 levels), hip abductor (4 levels), ankle plantarflexors (2 levels), and ankle dorsiflexors (2 levels). The balance retraining exercises consist of the following: knee bends (4 levels), backwards walking (2 levels), walking and turning around (2 levels), sideways walking (2 levels), tandem stance (2 levels), tandem walk (2 levels), one leg stand (3 levels), heel walking (2 levels), toe walking (2 levels), heel toe walking backwards (1 level), and sit to stand (4 levels).

Licensed physical therapists will deliver the OEP after a standard training session with the research team. For each OEP participant, a physical therapist will visit the home and prescribe a set of suitable exercises from the OEP manual. The same physical therapist will return bi-weekly three additional times to make progressive adjustments to the exercise protocol according to the OEP manual. Each of these four visits in the first 2 months will take approximately 1 hour. The physical therapist’s fifth visit will occur 6 months after the initial visit. During this last visit, the physical therapist will check that the OEP exercises are being done correctly and will also encourage the participant to continue with the exercise program. Overall, the participant is asked to perform the OEP balance and strength retraining exercises three times per week (approximately 30 minutes). In addition to the OEP manual, which contains a picture and description of each exercise, each participant will be provided with an adjustable cuff weight (in 0.9 kg increments; range = 0.9 to 9 kg) to be used with the OEP strength training exercises. Based on data from our proof-of-concept study [15], the OEP is safe for our target population; only 2 of the 36 OEP participants reported low back pain as adverse events.

#### Standard of care

Participants randomized to “standard of care” they receive standard of care – visits with a geriatrician.

### Adverse events monitoring

A physician and a statistician external to the daily activities of this study will review and compile a report for all adverse events reported in the study on a monthly basis. They will stop the study if the adverse event data demonstrate any hazards of the intervention (for example, increased falls or fracture) based on the monthly report.

### Statistical analyses

Our primary, secondary, and tertiary analyses will follow the intention-to-treat principle (that is, all individuals will be analyzed according to their group allocation regardless of compliance).

#### Primary outcome

The rate of falls (the primary outcome) will be compared between the two groups using a negative binomial regression model. The treatment assignment and stratification factors will be included in the model as covariates. Point and interval estimates for the rate ratio will be determined.

#### Secondary outcomes

We will conduct exploratory analyses on the secondary outcomes (PPA, TUG test and Short Performance Physical Battery). Given that a potential source of bias in this trial will result from patients being unblinded to their group allocation, group will be controlled for in all secondary analyses.

#### Economic evaluation

Our economic evaluation will examine the incremental costs and benefits generated by using the OEP intervention versus standard of care. The outcome of our cost effectiveness analysis is the incremental cost-effective ratio (ICER). By definition, an ICER is the difference between the mean costs of providing the competing interventions divided by the difference in effectiveness, where ICER = ∆cost/∆effect [62]. Both a cost-effectiveness analysis and a cost utility analysis will be performed. Based on the primary outcome of the RCT, we will determine the incremental cost of the OEP intervention per fall avoided, relative to standard treatment. We will also conduct a cost-utility analysis. In a cost-utility analysis, the primary outcome is the quality-adjusted life years. These are calculated based on the quality of life of a patient (measured using health utilities) in a given health state and the time spent in that health state. An important aspect of economic evaluations conducted alongside an RCT is how to deal with missing data due to attrition. We will follow recommendations by Oostenbrick and colleagues [63] and Briggs and colleagues [64], and the International Society for Pharmacoeconomics and Outcomes Research [65], in dealing with missing cost and effectiveness data. We will use a combination of imputation and bootstrapping to quantify uncertainty due to missing values.

#### Mediation analysis

We will use path analysis – a special case of structural equation modeling where all variables are observed – to investigate how physiological function and cognitive function mediate the effect of the intervention on the primary outcome (that is, falls). Using Mplus 5.1 (www.statmodel.com) we will fit a negative binomial regression model that includes one independent variable and mediator variables.

## Discussion

Our interdisciplinary research team will use a multi-pronged approach to explore the utility of OEP among seniors at high risk of future falls. The proposed trial may have important public health, economic, and mechanistic implications.

### Public health

The simple and proven exercise program (that is, the OEP) has already been implemented nationally in New Zealand. Therefore, if our study demonstrates the OEP is an efficacious and efficient (that is, cost-effective) secondary falls prevention program, our findings could be rolled out immediately by policy makers.

### Economic

The parallel economic evaluation is particularly important because, if the intervention proved to be cost-effective compared with standard of care, it would provide a strong argument for the OEP in the target population even at a time of fiscal restraint. We highlight that this intervention, the OEP, already has manuals, websites, and educational material ready for a ‘turn-key’ operation.

### Mechanistic

Better understanding of the primary mechanisms underlying the OEP (that is, our tertiary research objective) would increase our capacity to refine and develop novel interventions for secondary falls prevention for the aging population. If improved executive functions prove to play a significant role in falls reduction, it would be a major contribution to knowledge in this field.

## Trial status

As of 1 December 2014 we have obtained ethical approval, have registered the trial and we have successfully recruited 227 participants. We will aim to complete recruitment by 2017. One hundred and fifty four patients have completed 6-month follow-up, 133 have completed 12-month follow-up and 26 participants have dropped out. The median number of participation days for individuals who dropped out of the study was 103.5.

## Abbreviations

ABC: Activities-Specific Balance Confidence
EQ-5D-3L: Euro-Qol 5D three level version
ICER: Incremental cost-effective ratio
MMSE: Mini-Mental State Examination
MoCA: Montreal Cognitive Assessment
OEP: Otago Exercise Program
PPA: Physiological Profile Assessment
RCT: Randomized controlled trial
TUG: Timed Up and Go
